# Superficial Candidiasis: Cluster Analysis of Species Distribution and Their Antifungal Susceptibility In Vitro

**DOI:** 10.3390/jof11050338

**Published:** 2025-04-25

**Authors:** Marina Ranđelović, Aleksandra Ignjatović, Milica Đorđević, Maša Golubović, Marko Stalević, Nataša Rančić, Suzana Otašević

**Affiliations:** 1 Department of Microbiology and Immunology, Medical Faculty, University of Niš, 18000 Niš, Serbia; milica.djordjevic.mikrobiolog@medfak.ni.ac.rs (M.Đ.); otasevicsuzana@gmail.com (S.O.); 2 Centre of Microbiology, Public Health Institute Niš, 18000 Niš, Serbia; 3 Department of Medical Statistics and Informatics, Medical Faculty, University of Niš, 18000 Niš, Serbia; aleksandra.ignjatovic@medfak.ni.ac.rs; 4 Department of Dermatology, Clinical Center, University of Niš, 18000 Niš, Serbia; masabinic@gmail.com; 5 Department of Physiology, Medical Faculty, University of Priština—Kosovska Mitrovica, 38000 Kosovska Mitrovica, Serbia; 6 Doctoral Studies, Faculty of Medicine, University of Niš, 18000 Niš, Serbia; 7 Department of Epidemiology, Medical Faculty, University of Niš, 18000 Niš, Serbia; natasa.rancic@medfak.ni.ac.rs

**Keywords:** superficial candidiasis, cluster analysis, antifungal susceptibility

## Abstract

Background: Superficial candidiasis (SC) is widespread in humans worldwide. This study aimed to evaluate species distribution patterns and antifungal susceptibility through cluster analysis. Methods: A total of 180 *Candida* strains isolated from skin and nail samples of 1593 examined patients with suspected superficial fungal infection were identified by Matrix-assisted laser desorption in ionization-time of flight mass spectrometry (MALDI-TOF MS; Zybio EXS2600, China). Antifungal susceptibility was assessed using the commercial Integral System YEASTS Plus test (ISYPT; Liofilchem^®^, Italy). Agglomerative hierarchical cluster analysis was used to analyze species distribution and susceptibility. Results: *Candida parapsilosis* (44.4%) and *C. albicans* (40%) were the most prevalent causative agents of SC. Cluster analysis established two defined clusters. Cluster 1 (121 isolates) showed a statistically significant difference compared to Cluster 2 (54 isolates) in species distribution (*C. albicans* was dominant in the first and *C. parapsilosis* in the second cluster) as well as in susceptibility to ECN (*p* ≤ 0.001), KCA (*p* = 0.030), CLO (*p* ≤ 0.001), MCZ (*p* ≤ 0.001), ITZ (*p* ≤ 0.001), and FLU (*p* ≤ 0.006). Conclusion: The fact that one-third of isolates exhibited low sensitivity to antifungals highlights the need for a new approach in SC treatment, emphasizing the importance of mycological analyses, including in vitro testing of antifungal effectiveness.

## 1. Introduction

Superficial fungal infections (SFI) are among the most common human infections globally, affecting approximately 20–25% of the population [1]. In addition to dermatophytes and *Malassezia* species, yeasts from the genus *Candida* are also widespread as causative agents of SFI [2]. Based on published data, the dominant species of this genus that cause superficial candidiasis (SC) are *C. albicans* and *C. parapsilosis* complex.

Recent phylogenetic studies of the genus *Candida* have led to the reclassification and exclusion of several prevalent non-*albicans Candida* (NAC) species, such as *Candida* (*C.*) *krusei*, *C. glabrata*, *C. guilliermondii*, and *C. lusitaniae*, which are now classified under the genera *Pichia*, *Nakaseomyces*, *Meyerozyma*, and *Clavispora*, respectively [3]. However, many experts in the field of medical mycology suggest that this new classification could create confusion and dilemmas in interpreting mycological analysis results. This is the reason why the widely accepted approach is to continue using the old nomenclature, which we also applied when listing isolated *Candida* yeasts [4].

Superficial candidiasis includes skin infections as well as infections of the nails and surrounding tissues (onychomycosis and paronychia). *Candida* skin infections commonly occur in intertriginous areas, such as spaces between skin folds, fingers, under the breasts, in the armpits, and in the groin [5]. Several factors can predispose individuals to these infections, including humidity, heat, friction, and skin maceration. The populations most sensitive to SC are newborns, older people, and immunosuppressed individuals [6,7]. Typical lesions associated with SC include erythematous plaques, skin desquamation with satellite pustules, and a tendency for the infection to spread to healthy skin. Candidosis affecting the nail plate and surrounding tissues is recognized as a distinct clinical entity known as *Candida* onychomycosis [8]. This condition often arises as an occupational disease or in individuals whose hands frequently come into contact with sugars or harsh cleaning and disinfecting agents in humid environments [9]. It typically presents as painful erythematous swelling around the nails (perionychium). If untreated, this infection may lead to nail plate deformation and potentially result in the complete destruction of the nail over time [10].

The diagnosis of SC, like other superficial fungal infections, is primarily based on clinical findings (empirical approach) and often involves microscopic examination for initial diagnosis. Although very rarely, laboratory culture and identification of the causative agent, along with testing its antifungal susceptibility, may also be conducted [11]. Identifying the *Candida* species is crucial since not all species respond equally to commonly used antifungal medications. While topical antifungal agents are frequently applied to treat SC, systemic antifungal drugs may be necessary, particularly in cases of onychomycosis. As most of the SC cases are managed without laboratory confirmation, this study aimed to evaluate the mycological findings (including species identification and in vitro antifungal susceptibility patterns) using cluster analysis.

## 2. Materials and Methods

This research was designed as a prospective study conducted in the Center for Microbiology, Institute for Public Health, Nis. The IRB decision was unnecessary for this survey since specimens were examined in vitro. From the beginning of 2022 to the end of 2024, among 1593 examined patients, superficial candidiasis was clinically and laboratory confirmed in 180. All isolates of *Candida* spp. were differentiated using a matrix assisted laser desorption ionization time of flight (MALDI TOF) mass spectrometry, and their sensitivity to antifungal drugs was examined in vitro.

### 2.1. Microbiological Analysis

For the cultivation of *Candida* strains, Sabouraud dextrose agar (SDA) and chromogenic *Candida* medium (Liofichem^®^, Roseto degli Abruzzi, Italy) were used. The incubation time was up to 7 days at 37 °C and 28 °C.

Cultivated yeast species were identified using MALDI-TOF MS (Zybio EXS2600, Chongqing, China). Briefly, the procedure of this method included picking a colony of the tested strain from SDA using a thin wooden stick and transferring it to one of the 96 fields of the MALDI metal plate. The thin layer of sample applied was dried at room temperature and then covered with 1 μL of 70% formic acid. After drying, 1 μL of matrix solution (α-Cyano-4-hydroxycinnamic acid) was added. When all the prepared samples were dry, the plate was placed in the MALDI-TOF mass spectrometer. The MALDI-TOF MS was calibrated and validated once a week with a bacterial test standard according to the manufacturer’s instructions. Scores of 2.0 or higher indicate reliable identification at the species level. Scores between 1.7 and 2.0 indicate reliable identification at the genus level, while scores below 1.7 indicate no reliable identification [12].

### 2.2. Antifungal Susceptibility Testing

Antifungal susceptibility testing was determined using the commercial Integral System YEASTS Plus (ISYP) test (Liofilchem^®^, Italy). This commercial test allows testing the effectiveness of antimycotics of the polyene group [Nystatin (NY at a concentration of 1.25 μg/mL) and Amphotericin B (AMB at a concentration of 2 μg/mL)]; antimetabolite flucytosine (FCY at a concentration of 16 μg/mL); groups of imidazole derivatives [Econazole (ECN at a concentration of 2 μg/mL); Ketoconazole (KCA at a concentration of 0.5 μg/mL); Clotrimazole (CLO at a concentration of 1 μg/mL); Miconazole (MCZ at a concentration of 2 μg/mL)]; as well as triazole groups [Itraconazole (ITZ at a concentration of 1 μg/mL); Voriconazole (VOR at a concentration of 2 μg/mL) and Fluconazole (FLU at a concentration of 64 μg/mL)]. Susceptibility of isolates to the tested antifungals was interpreted as S—sensitive to the concentration of tested antimycotic in ISYP test; I/R (intermediate sensitivity/resistance)—lower sensitivity to the concentration of tested antimycotic in ISYP test, based on the manufacturer’s instructions and following the recommendations of CLSI (The Clinical and Laboratory Standards Institute—Subcommittee on Antifungal Susceptibility Testing—Institute for Clinical and Laboratory Standards, subcommittee for Testing the Sensitivity of Fungi to antifungals) [13]. *Candida parapsilosis* ATCC 22019 and *C. krusei* ATCC 6258 were strains used for quality control.

### 2.3. Statistics

Agglomerative hierarchical cluster analysis of ten antimycotics as binary variables was performed. In the first step, outliers in clustering were detected and removed, and the final cleaned dataset consisted of 175 observations. The Jaccard distance was used on the binary dataset, and hierarchical cluster analysis with Ward’s method. The D method was applied. The dendrogram was used to visualize cluster separation. The optimal number of clusters was determined using the elbow and silhouette methods. The cluster analysis successfully identified two well-separated clusters in the dataset. Validation metrics (Silhouette method) confirmed a good clustering solution. A comparison of demographic and clinical characteristics between clusters was performed using the *t*-test, the Chi-squared test, and the Fisher test. The null hypothesis was tested at a significance level of *p* < 0.05. The statistical analysis was performed in R and R Studio by using packages factoextra and cluster [14].

## 3. Results

**Figure 1:**
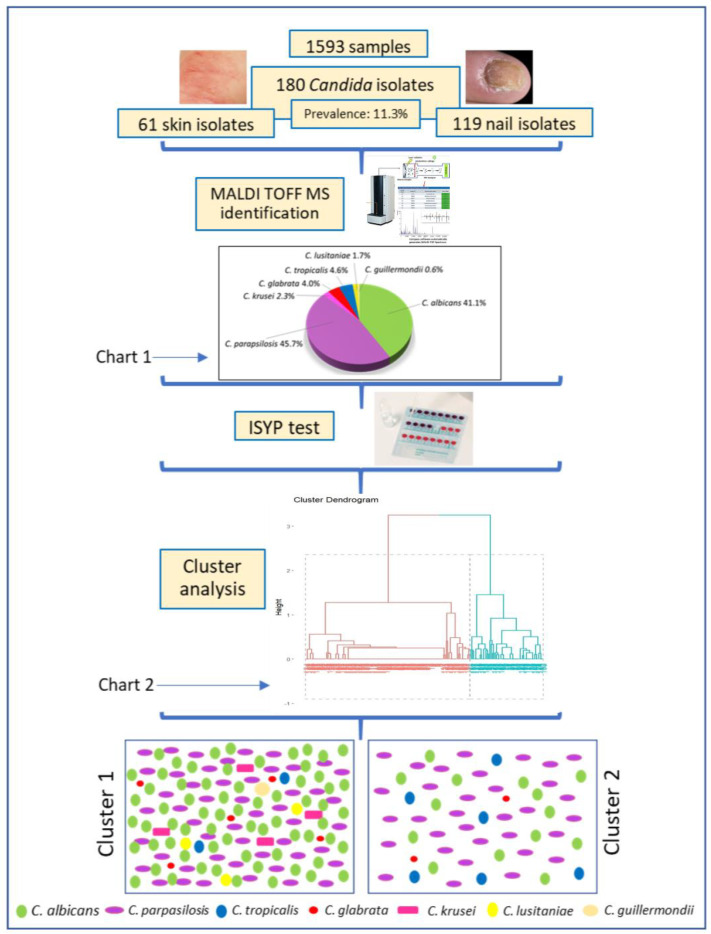
Schematic representation of study design. Statistically significant difference in the two clusters regarding yeast species: 47.9% of *Candida albicans* in Cluster 1; 59.3% of *Candida parapsilosis* in Cluster 2 (*p* = 0.003). MALDI-TOF MS—Matrix-assisted laser desorption in ionization-time of flight mass spectrometry, ISYP test—Integral System YEASTS Plus test.

Our study design and results are shown in Figure 1. Superficial candidiasis was proven in 180 of 1593 examined patients (prevalence 11.3%). All isolates of *Candida* spp. causative agents of SC (61 skin and 119 nail isolates) were mycologically and statistically analyzed.

The species *C. parapsilosis* was identified in the highest percentage (45.7%), followed by *C. albicans* (41.1%). The species *C. tropicalis* (4.6%), *C. glabrata* (4.6%), *C. krusei* (2.3%), *Candida lusitaniae* (1.7%), and *Candida guillermondii* (0.6%) were identified in significantly fewer cases (Chart 1 in Figure 1).

The evaluation of in vitro antifungal susceptibility results showed that *C. albicans* had very satisfactory susceptibility to all tested antifungals except polyenes ECN and CLO (Table 1). On the contrary, a significantly lower percentage of NAC species was sensitive to all included antimycotics. *Candida parapsilosis* was dominant among these strains, and isolates of this species were mainly susceptible to AMB, KCA, FCY, and triazoles. The least number of isolates was sensitive to ECN, followed by CLO, MCZ, and NY. Well-known resistant species such as *C. glabrata* showed low sensitivity to AMB, ECN, and CLO, while *C. krusei* strains were less susceptible to polyenes and FLU.

Only a few isolates of *C. tropicalis* were susceptible to the tested antimycotics, while lower sensitivity was determined for ECN and CLO (25%), ITZ (50%), and NY, MCZ, and FLU (62.5%). Strains of *C. lusitaniae* were sensitive to all tested antifungals except ECN, ITZ, and FLU.

In order to analyze the species distribution pattern of *Candida* spp. susceptibility to 10 tested antimycotics in vitro, cluster analysis was applied. This kind of analysis might identify patterns (clusters) of yeast species with shared characteristics, such as antifungal susceptibility, which is the case in this study. Before clustering, the dataset was standardized so that each variable (different antifungal sensitivity/resistance) contributed equally to the separation process. The optimal number of clusters was determined using the elbow and silhouette methods, which helped identify distinct groupings with minimal overlap. This statistical analysis of the data established the existence of two clusters (Chart 2 in Figure 1).

The first cluster (Cluster 1) consisted of 121 (69.1%) strains with a high percentage of isolates susceptible to most antifungal drugs, while the second cluster (Cluster 2) included 54 (30.9%) isolates that were highly susceptible to the tested antimycotics.

**Figure 2:**
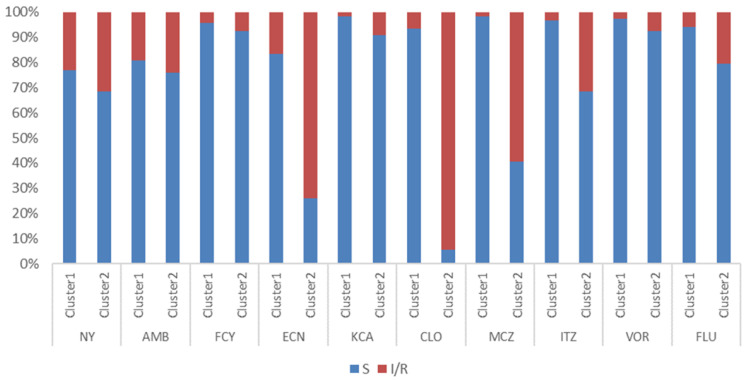
Comparison of isolates’ antifungal susceptibility from two clusters. NY—nystatin, AMB—amphotericin B, FCY—flucytosine, ECN—econazole, KCA—ketoconazole, CLO—clotrimazole, MCZ—miconazole, ITZ—itraconazole, VOR—voriconazole, FLU—fluconazole; Statistically significant difference in susceptibility to ECN (*p* ≤ 0.001), KCA (*p* = 0.030), CLO (*p* ≤ 0.001), MCZ (*p* ≤ 0.001), ITZ (*p* ≤ 0.001), and FLU (*p* ≤ 0.006); S—sensitive to the concentration of tested antimycotic in Integral system Yeasts plus test; I/R (intermediate sensitivity/resistance)—lower sensitivity to the concentration of tested antimycotic in Integral system Yeasts plus test.

Further analysis showed that the statistically significant difference in the two clusters was established regarding susceptibility to ECN (*p* ≤ 0.001), KCA (*p* = 0.030), CLO (*p* ≤ 0.001), MCZ (*p* ≤ 0.001), ITZ (*p* ≤ 0.001) and FLU (*p* = 0.006) (Figure 2). While Cluster 1 involved species that showed sensitivity to the following antifungals: ECN (83.5%), KCA (98.3%), CLO (93.4%), MCZ (%), (98.3%), ITZ (96.7%), FLU (94.2%) in high percentage (Figure 2), Cluster 2 included strains which were characterized by lower susceptibility to mentioned antimycotics.

However, no significant difference was proven between clusters regarding susceptibility to tested polyenes (NY, AMB), antimetabolite drugs (FCY), and VOR triazole (Figure 2).

Additionally, the distribution of yeast species significantly differed regarding clusters (*p* = 0.003). Namely, *C. albicans* (47.9%) was dominant in Cluster 1, while *C. parapsilosis* (59.3%) was the most prevalent in Cluster 2 (Table 2).

By comparing patient characteristics as well as the season of infection occurrence by clusters, no differences were found in relation to patients’ gender (*p* = 0.640) or age (*p* = 0.354), and the infection did not have a seasonal character (*p* = 0.967) (Table 2).

## 4. Discussion

In recent years, the prevalence of dermatomycosis and onychomycosis due to *Candida* spp. infection significantly increased. This prevalence ranged from 16.2% in Iran to 22.3% in China to even 80% in Brazil [15,16,17]. In Serbia, based on the evaluation of data over the last 40 years, it can be pointed out that *Candida* spp. has been a causative agent of skin and nail fungal infections in approximately 10% of examined patients [2]. Moreover, in a group of patients with clinical and laboratory-determined SFI, *Candida* spp. started to predominate as causative agents, with a prevalence of 30% in patients with SFI of skin and nails until 2017 [2], to 60.1% in the period from 2019 to 2022 [18]. This study highlights that in the last three years, SC prevalence has maintained the same level, slightly elevating to 11.3%. Even with applied MALDI-TOF mass spectrometry, the spectra of *Candida* species have not changed significantly. The trend of higher representation of NAC species continued with the domination of *C. parapsilosis* [18]. The reason for the lower prevalence of skin and nail *C. albicans* infection related to *C. albicans* mucosal infection could be that the primary virulence factor of *C. albicans* is its ability to undergo dimorphic growth, transitioning from a unicellular (yeast form) to a multicellular, mycelial form. Environmental factors influence the expression of this virulence factor, and it is well known that temperatures higher than 37 °C and high concentrations of CO_2_ (present on mucous membranes but not on the skin and nails), along with other conditions, promote filamentation, which can be considered as the first link in the pathogenesis chain [19]. Other isolated NAC species included *C. glabrata*, *C. tropicalis*, *C. krusei*, *and C. guilliermondii*, which were isolated in our previous investigation [2]. However, besides the mentioned strains, *C. lusitaniae* was also isolated in the present study. The range of different species correlates primarily with the applied methodology, i.e., tests for yeast differentiation [20], as well as with the region where the research was done. In some studies in Brazil and China, *C. albicans* (˃90%) was shown to be the most common causative agent of SC [16,21]. On the contrary, investigation in Turkey obtained completely opposite results, where *C. glabrata* (52.54%) was the most prevalent species, followed by *C. guillermondii* (15.25%) and *C. albicans* (11.86%). The low percentage of isolated *C. albicans* strains showed a significant shift from *C. albicans* to NAC species in the case of SC [22]. Although the prevalence of *C. albicans* and NAC species varies in different studies, the spectrum of isolated yeasts is generally similar. It includes the same species isolated in our region, with sporadic detection of *C. dubliniensis*, *C. zeylanoides*, and *C. famata* at a low percentage. Additionally, molecular testing provided differentiation of *C. parapsilosis* complex, and *C. parapsilosis sensu stricto*, *C. orthopsilosis*, and *C. metapsilosis* were identified as causative agents of SC [23].

The local treatment, which is preferable to systemic antifungal drug therapy, for onychomycosis involves recommended amorolfine HCl 5% acrylic, ciclopirox 8% acrylic, and terbinafine (78.22 mg terbinafine/mL) nail polish, which is applied in accordance with the dermatological findings. In the case of *Candida* onychomycosis, ciclopirox or amorolfine for topical nail polish treatment is recommended [24]. To date, intermittent long-term therapy with low-dose terbinafine has been the first choice for systemic treatment. This antimycotic has a primarily fungicidal effect due to squalene epoxidase inhibition, which leads to ergosterol deficiency. It was determined that terbinafine had significantly higher mycological healing rates compared to ITZ [24]. However, in areas where this allylamine is unavailable, ITZ could be used as continuous or pulse systemic treatment for onychomycosis. Another possibility for pulse treatment is the oral use of FLU. Recommendations for the systemic therapy of onychomycosis caused by *C. albicans* and *C. parapsilosis* include FLU (continuous or interval therapy), ITZ, or terbinafine [24]. On the other hand, polyenes (NY), imidazoles (KCA, ECN, fenticonazole, oxiconazole, etc.), allylamines (naftifine, terbinafine), benzylamines (butenafine), and hydroxy pyridone-ciclopirox can be used in the treatment of skin SC [25,26]. Clotrimazole, NY, and MCZ are the most commonly used among the recommended antifungals for local application [27]. Based on the published results, no difference in effectiveness was noted among the mentioned antifungals in the treatment of patients with SC, and it was determined that this effectiveness was in the range of 73–100% [25]. In addition to the mentioned antifungal drugs, in recent years, fenticonazole has been recommended as a successful antifungal in the treatment of *Candida* dermatitis, and the satisfactory effect of allylamine has also been recorded [28]. As for systemic therapy, FLU and ITZ are recommended in severe or chronic skin SC. Most of the published data on the effectiveness of antifungal drugs were obtained in clinical studies that tested the effect of these drugs in vivo. This practically means that the success of antifungal treatment was determined based on the monitoring of patients with SFI. However, with the increasing prevalence of SC in recent years, more research has been aimed at determining the *Candida* species and examining the antifungal susceptibility in vitro. With the same goal in our study, we examined the isolated *Candida* species and their sensitivity in vitro to 10 antifungals. For a more straightforward evaluation of data, we used cluster analysis. This statistical method is a technique intensively used in medicine to identify patterns of symptoms and clinical signs for easier definition of diagnostic categories [29,30]. Namely, this statistical method is increasingly used when processing a large amount of data [31]. In this study, it was used to classify and group several different causative agents of SC, as well as their sensitivity to 10 investigated antifungal drugs.

Applied statistical method separated and defined two clusters, where cluster 1 involved more than two-thirds of isolates (69.1%) with statistically significant difference (*p* = 0.003) in the representation of *C. albicans* (whose prevalence was 47.9% in Cluster 1 and 25.9% in Cluster 2). Conversely, NAC species, primarily *C. parapsilosis*, were the dominant species in cluster 2 with a prevalence of approximately 60%. Further, a high percentage of the Cluster 1 members were susceptible to the most antifungal drugs (ECN-83.5%, KCA-98.3%, CLO-93.4%, MCZ-98.3%, ITR-96.7%, FLU-94.2%). However, the second cluster included 30.9% strains with low susceptibility to tested antimycotics, and a statistically significant difference was established regarding susceptibility to ECN (*p* ≤ 0.001), KCA (*p* = 0.030), CLO (*p* ≤ 0.001), MCZ (*p* ≤ 0.001), ITZ (*p* ≤ 0.001) and FLU (*p* = 0.006) (Figure 1). Most yeast isolates from both clusters were sensitive to the tested polyenes, FCY, and VOR.

Numerous studies have investigated the susceptibility of *Candida* strains, causative agents of SC, to antimycotics using various methods such as the broth microdilution method, disk diffusion, E tests, and some commercial antimycogram tests. In terms of susceptibility to polyenes, most of the investigated yeasts, regardless of the species, showed satisfactory susceptibility to NY and AMB [16,21,32,33]. In our study, *C. glabrata* strains were notably less susceptible (42.9%) than other species. Similar results with our research were also noted in susceptibility to 5-FCY with high sensitivity rates to this antimycotic [2,16]. Among imidazoles, MCZ was the most frequently investigated as an SC treatment option, and the highest resistance rate to this antimycotic was observed in *C. albicans* strains. In the study conducted by Bilal et al., 30% of *C. albicans* strains were resistant to MCZ [16], while Abastabar et al. recorded low susceptibility to MCZ in more than half of all tested *C. albicans* strains [34]. In the same study, notably better susceptibility was observed for NAC strains. These results differ from our findings because even 90.3% of *C. albicans* strains were susceptible to MCZ, and around a third of the strains of *C. parapsilosis*, *C. glabrata*, and *C. tropicalis* showed lower susceptibility to this antifungal agent. Another imidazole with lower efficacy on the strains from the second cluster was KCA. Still, despite this statistically significant difference, it must be pointed out that this antimycotic showed efficacy in isolates ranging from 87.5% to 100%. Based on published data, this antimycotic had generally satisfying effects on yeast, the causative agent of SC [16,23]. When considering the sensitivity of these strains to triazoles, varied results were obtained by different authors. In studies in which most isolates were *C. albicans* and *C. parapsilosis*, as in our research (86.7%), these species were highly susceptible to FLU, ITZ, and VOR [2,33,35,36]. On the other hand, the most resistant species to this class of antifungal agents appears to be *C. glabrata* [22,35], but also, contrary to previously mentioned research, *C. albicans* as well. In a study by Crocco et al., 14.5% of *C. albicans* strains were resistant to ITZ [21], while this value was 8.6% in the study by Mohammadi et al. [32]. Abastabar et al. recorded that even 69% of *C. albicans* strains were resistant to this antimycotic [34]. In the study by Kurç et al., in which the majority of examined species were *C. glabrata* (52.54%), *C. guillermondii* (15.25%), and *C. albicans* (11.86%), more than a third of all examined yeast strains were resistant to ITZ [22]. In our study, the most resistant species to ITZ was *C. tropicalis* (50%), followed by *C. lusitaniae* (33.3%) and *C. glabrata* (28.6%), while the majority of *C. albicans* (93.1%) and *C. parapsilosis* (88.8%) strains were susceptible to this antimycotic. As already mentioned, in many studies, the successful effect of FLU on causative agents of SC in vitro was proven. However, it should be noted that this antifungal was less effective against *C. albicans* strains in some cases. Namely, Abastabar et al. recorded 57% [34], and Mohammadi et al. 11.4% *C. albicans* isolates resistant to FLU [32]. Although yeast causative agents of SC are generally proven to be sensitive to VOR, in the study by Bilal et al., 38.79% of *C. albicans* strains showed lower sensitivity to this antimycotic [16]. In our study, strains of all species were susceptible to VOR, with the exception of 25% of *C. tropicalis* isolates.

Although other studies have found a statistically significant difference regarding gender and age [37], we did not prove the difference when analyzing demographic characteristics in this investigation using cluster analysis. However, based on public data, more precisely, different results and specific gender or age predisposition for the onset of SC cannot be established. Although it could be expected that a warmer climate during the summer months may result in a more frequent occurrence of SC, the seasonal character of this infection was not observed in our research.

## 5. Conclusions

To conclude, it can be pointed out that SC of skin and nails is a highly prevalent fungal infection worldwide. Recently, NAC species have become an increasingly dominant cause of these infections, and there are many reports of the higher occurrence of resistant strains. Cluster analysis is an excellent statistical method for summarizing large data. By applying this analysis, it is easier to evaluate the obtained results as well as to define the scale of the problem. Establishing one-third of *Candida* isolates with low sensitivity to the most commonly used antifungals, in this case, imposes a new approach in SC therapy. This implies the need for mycological analyses that would also include the examination of antimycotic efficiency in vitro. Although good efficacy in vitro is not always followed by successful healing, the general attitude is that resistance established in vitro is certainly confirmed during treatment.

## Figures and Tables

**Table 1:** Percentage of sensitive isolates to tested antimycotics.

Species/No	NY	AMB	FCY	ECN	KCA	CLO	MCZ	ITZ	VOR	FLU
*C. albicans*/72	70.8	73.6	94.4	83.3	94.4	70.8	90.3	93.1	97.2	94.4
NAC spp./103	76.7	83.5	95.1	53.4	97.1	63.1	73.8	84.5	95.1	86.4
*C. parapsilosis*/80	76.3	85.0	96.3	53.8	97.5	63.8	72.5	88.8	96.3	91.3
*C. tropicalis*/8	62.5	87.5	87.5	25.0	87.5	25.0	62.5	50.0	75.0	62.5
*C. glabrata*/7	85.7	57.1	85.7	42.9	100.0	57.1	71.4	71.4	100.0	100.0
*C. krusei*/4	75.0	75.0	100.0	100.0	100.0	100.0	100.0	100.0	100.0	25.0
*C. lusitaniae*/3	100.0	100.0	100.0	66.7	100.0	100.0	100.0	66.7	100.0	66.7
*C. guillermondii*/1	100.0	100.0	100.0	100.0	100.0	100.0	100.0	100.0	100.0	100.0

NY—nystatin, AMB—amphotericin B, FCY—flucytosine, ECN—econazole, KCA—ketoconazole, CLO—clotrimazole, MCZ—miconazole, ITZ—itraconazole, VOR—voriconazole, FLU—fluconazole; NAC—non-*albicans Candida* species.

**Table 2:** Demographic characteristics of patients and species distribution pattern in clusters.

		Total		Cluster 1		Cluster 2		*p*
		*n*	%	*n*	%	*n*	%	
Age †		47.04 ± 22.48		45.80 ± 23.44		49.81 ± 20.10		0.354 ^1^
Gender	Male	43	24.6	28	23.1	15	27.8	0.640 ^2^
	Female	132	75.4	93	76.9	39	72.2	
Season	Winter	48	27.4	34	28.1	14	25.9	0.967 ^2^
	Spring	50	28.6	35	28.9	15	27.8	
	Summer	48	27.4	33	27.3	15	27.8	
	Autumn	29	16.6	19	15.7	10	18.5	
Cause	*Candida parapsilosis*	80	45.7	48	39.7	32	59.3	0.003 ^2^
	*Candida albicans*	72	41.1	58	47.9	14	25.9	
	*Candida tropicalis*	8	4.6	2	1.7	6	11.1	
	*Candida glabrata*	7	4	5	4.1	2	3.7	
	*Candida krusei*	4	2.3	4	3.3	0	0	
	*Candida lusitaniae*	3	1.7	3	2.5	0	0	
	*Candida guillermondii*	1	0.6	1	0.8	0	0	

^1^ *t*-test, ^2^ Chi-squared test, † Mean ± Standard deviation.

## Data Availability

The original contributions presented in this study are included in the article. Further inquiries can be directed to the corresponding author.
